# The ADHD advantage: Comorbid attention-deficit/hyperactivity disorder is associated with earlier diagnosis and better academic outcomes in children with specific language disorder

**DOI:** 10.1371/journal.pone.0356489

**Published:** 2026-10-05

**Authors:** Manas Soongprasit, Nuthawadee Tiachunpun, Nitchawan Jongrakthanakij, Piangjai Tongpoung, Masatha Thongpan

**Affiliations:** Department of Psychiatry, Faculty of Medicine Ramathibodi Hospital, Mahidol University, Bangkok, Thailand; IRCCS Medea: Istituto di Ricovero e Cura a Carattere Scientifico Eugenio Medea, ITALY

## Abstract

Specific learning disorder (SLD) is a neurodevelopmental disorder characterized by difficulties in reading, writing, and mathematics despite normal intelligence. One of the most common comorbidity of SLD is attention-deficit hyperactivity disorder (ADHD). However, differences between patients with SLD alone and SLD with ADHD remain underinvestigated. This study aims to compare clinical and psychosocial characteristics between these two groups. This retrospective study analyzes pediatric psychiatric patient data from Research Electronic Data Capture (REDCap) at the Learning Disorder Outpatient Clinic, Ramathibodi Hospital, Thailand from 2017 to 2023. Chi-square tests, t-tests, and mediation analyses were used to examine group differences and causal relationships among significant factors. Among 251 patients, the SLD with ADHD group exhibited less severe delays in reading (p = 0.003), writing (p = 0.001), and math (p = 0.007). This group also visited the clinic earlier (median: grade 3) than the SLD alone group (median: grade 5). Mediation analysis revealed that ADHD had a direct impact on reading and math delays (coefficients: 0.47, 0.38). No significant differences were found in gender, IQ, birth risks, parental education, or family income. Early detection explained the advantage in writing skills but not fully for reading and math, suggesting that other neurocognitive factors may be involved. Therefore, early recognition and referral should be encouraged to promote better academic outcomes.

## Introduction

Specific learning disorder (SLD), as defined by the DSM-5, is a neurodevelopmental condition characterized by persistent difficulties in one or more key academic areas (reading, writing, or mathematics) despite adequate education and normal intellection. SLD is not explained by intellectual disability or global developmental delay. Affected individuals are expected to have intellectual functioning within normal range [1]. The condition includes several subtypes based on areas of impairment. For reading, deficits may involve word reading accuracy, decoding, reading fluency, or comprehension. Writing challenges include spelling, grammar, and written expression. Mathematics difficulties may relate to number sense, calculation, or mathematical reasoning. Among these, reading disorders are the most common, followed by written language and mathematics disorders. All of which show high rates of comorbidity with attention-deficit/hyperactivity disorder [1].

Attention-deficit/hyperactivity disorder (ADHD) is a common neurodevelopmental disorder characterized by persistent symptoms of inattention, hyperactivity, and impulsivity that interfere with a child’s functioning across multiple settings such as home and school [1]. According to the DSM-5, symptoms must be present before the age of 12, persist for at least six months, and occur in at least two settings. Children with ADHD may struggle to sustain attention, follow instructions, or remain seated. They often display impulsive or overly active behaviors. These difficulties can significantly impact academic performance, social interactions, and family relationships. In some cases, children may also exhibit externalizing behaviors such as aggression, oppositional defiance, or conduct problems, increasing their risk for grade retention, placement in special education, or school dropout [2]. ADHD is also highly comorbid with learning disorders, and its long-term effects may impact health, behavior, and emotional development [1].

The comorbidity of SLD and ADHD is relatively high, with approximately 31% to 45% of students with ADHD having SLD and vice versa [3]. Although estimates vary widely depending on the criteria used to define SLD [4], children with ADHD are roughly three to four times more likely to exhibit SLD than their typically developing peers [4].

Both disorders are found to have a shared etiological pathway. First, the association between ADHD and achievement difficulties is driven more by inattentive than hyperactive-impulsive symptoms, especially among children of elementary school age (6–11 years old) [2]. Second, children with ADHD and SLD have overlapping deficits in working memory and processing speed. Third, several genetic studies implicate that multiple genes are involved in both ADHD and reading disabilities [4].

While both disorders affect academic achievement, they do so via different mechanisms. SLD results in direct academic impairments in reading, writing, or mathematics. In contrast, ADHD interferes with academic success through difficulties in sustaining attention, memory deficits, and disruptive classroom behavior [4].

Although they share etiologies, different clinical characteristics can still be observed. For instance, the comorbid group often shows greater deficits in response inhibition and processing speed than the SLD alone group [1].

However, previous studies rarely compare these groups directly across broader academic and cognitive domains. Therefore, this study aims to compare the characteristics of children with SLD alone and those with SLD and comorbid ADHD across literacy skills (reading, writing, mathematics), cognitive ability (IQ), and risk factors (e.g., birth complications, family education, and income). We hypothesize that children with both SLD and ADHD will exhibit more severe academic difficulties and worse risk profiles than those with SLD alone. This could help guide more tailored intervention strategies.

## Methods

### Study design

This retrospective cross-sectional study was conducted at the Learning Disorder Outpatient Clinic, Department of Psychiatry, Ramathibodi Hospital, Mahidol University. Data was extracted from the Psychiatric Patients Data Registry (PPDR) using the Research Electronic Data Capture (REDCap) system covering from January 2017 to December 2023. Ethical approval was obtained from the Institutional Review Board of Ramathibodi Hospital (MURA 2025/459).

### Participants

Children diagnosed with specific learning disorder (SLD) either alone or with comorbid attention-deficit/hyperactivity disorder (ADHD) were included in the study. All participants had been formally issued certificates for SLD by a child and adolescent psychiatrist and had completed IQ assessments using the third edition of Wechsler Intelligence Scale for Children (WISC-III). Inclusion criteria required complete data on IQ testing and a standardized achievement test by a multidisciplinary team comprising psychologists. Children with neurological disorders, motor or sensory impairments, language barriers, or other conditions that could interfere with test performance were excluded. Written informed consent was obtained from parents or guardians prior to inclusion in the dataset.

### Measures

Demographic data was collected through caregiver interviews and intake forms, including age, gender, grade level at first clinic visit, parental education, and family income. Birth risk information was obtained from patient history, including the presence of prematurity, low birth weight, or neonatal complications. Each factor was coded as 0 (absent) or 1 (present), resulting in a total birth risk score ranging from 0 to 3. Cognitive functioning was assessed using the Thai version of the third edition of the Wechsler Intelligence Scale for Children (WISC-III). Full-scale IQ scores were obtained and used for analysis.

The severity of learning disorder was evaluated using an academic achievement Test called Wide Range Achievements Test (WRAT-Thai) [5]. The test consisted of 3 dimensions including writing, reading and math. The writing section consisted of 50 questions, in which the participants were required to write the words as dictated onto the given blank space. The reading section consisted of 60 questions, in which the participants were to read the selected passage out loud. Lastly, for the math section, consisting of 44 questions, the participants were to solve mathematical problems and write down answers on the given blank space. All the tests employed in this research were done by clinical psychologists at Ramathibodi Hospital and required approximately one hour to complete. Results were reported as grade-level delays in reading, writing, and mathematics. For example, a score of −3 indicates performance three grades below the expected level.

### Statistical analysis

Statistical analyses were conducted using Stata software (version 18). Descriptive statistics summarized characteristics of the participants. Continuous variables were reported as means and standard deviations. Categorical variables were reported as counts and percentages. Group comparisons between children with SLD alone and those with comorbid ADHD were conducted using independent samples t-tests for continuous variables and chi-square tests for categorical variables.

Linear regression analysis was used to identify factors associated with the severity of SLD. Finally, mediation analyses were conducted to explore whether early detection, defined as early grade level at first OPD visit, mediated the relationship between ADHD status and academic performance in reading, writing, and math. A p-value of < 0.05 was considered statistically significant.

## Results

A total of 251 children were included: 88 in the SLD alone group and 163 in the SLD and comorbid ADHD group. The proportion of male participants was similar between groups (80.6% vs 78.5%, p = 0.688), indicating no significant gender difference. However, the median grade level at first outpatient visit was significantly lower in the SLD and ADHD group (median grade 3.66) compared to the SLD alone group (median grade 4.50), (p = 0.004), suggesting that children with comorbid ADHD were more likely to be detected and referred earlier. There was also no statistically significant difference in full IQ scores between the comorbid group compared to SLD alone group (103.21 vs. 99.29, p = 0.061). Similarly, differences in verbal and performance IQ scores were not significant. Regarding overall school performance, the fail/pass status, defined as whether the child had ever failed a school exam, did not significantly differ between the two groups (p = 0.725) (Table 1).

**Table 1:** Comparison of clinical characteristics between children with SLD alone and children with SLD and comorbid ADHD.

Total n = 251	SLD alone (n = 88)	SLD with comorbid ADHD (n = 163)	Statistical test	p-value
**Mean diff (95%CI)**
**Median grade level at first outpatient visit**	**4.50 ± 2.29**	**3.66 ± 2.02**	**0.84 ± 0.29 (0.26, 1.41)**	**0.0042**
Mean full IQ score	99.29 ± 15.56	103.21 ± 14.68	−3.92 ± 2.08 (−8.02, 1.77)	0.0607
Mean verbal IQ score	96.03 ± 15.64	100.16 ± 14.98	−4.13 ± 2.22 (−8.49, 0.24)	0.0638
Mean performance IQ score	103.76 ± 15.24	106.79 ± 15.50	−3.03 ± 2.46 (−7.46, 1.39)	0.1784
Mean birth risks score	0.63 ± 0.85	0.56 ± 0.84	0.076 ± 0.13 (−0.19, 0.34)	0.5661
Mean family risk score	0.29 ± 0.53	0.43 ± 0.61	−0.14 ± 0.14 (−0.42, 0.13)	0.3064
**Mean reading delay (in years relative to grade level)**	**−2.14 ± 1.47**	**−1.57 ± 1.24**	**−0.56 ± 0.19 (−0.93, −0.19)**	**0.003**
**Mean writing delay**	**−2.99 ± 1.51**	**−2.4 ± 1.16**	**−0.59 ± 0.18 (−0.95, −0.23)**	**0.01**
**Mean math delay**	**−1.62 ± 1.46**	**−1.13 ± 1.16**	**−0.49 ± 0.18 (0.85, −0.13)**	**0.007**
			Chi-square	
Male gender	71 (80.6%)	128 (78.5%)	0.1615	0.688
History of seizures	5 (5/63 = 7.9%)	12 (12/123 = 9.8%)	0.1661	0.684
Fail/Pass	6 (6/38 = 15.8%)	16 (16/97 = 18.4%)	0.1234	0.725

A cumulative risk score was calculated to increase the sensitivity between pre/perinatal risks and severity of neurodevelopmental delay [6]. One point was given for each of the following: birth risks (prematurity, low birth weight, neonatal complications), history of seizures, and history of speech delay, with a cumulative score ranging from 0 to 3. The mean birth risk score was slightly higher in the SLD alone group (0.63 vs 0.56), but not statistically significant (p = 0.566). Seizure history was reported in 7.9% of the SLD alone group and 9.8% of the SLD and ADHD group (p = 0.684). The family risk score (1 equals at least one family member with a history of learning difficulties) was slightly higher in the SLD and ADHD group (0.43 vs 0.29), but this difference was not significant (p = 0.306) (Table 1). Both findings suggest that early life or familial risk factors do not clearly explain the performance difference between our samples.

Achievement test results, expressed as grade level delays in academic years, revealed that children with SLD alone had significantly greater delays in all academic domains. The mean delay in reading was −2.14 years in the SLD alone group compared to −1.57 years in the SLD and comorbid ADHD group (p = 0.003). Similarly, the writing delay was −2.99 compared to −2.40 years (p = 0.001), and the math delay was −1.62 compared to −1.13 years (p = 0.007) (Table 1). These findings indicate that children with SLD and comorbid ADHD exhibited less severe academic delays than those with SLD alone, which is contrary to our initial hypothesis. This prompted further mediation analysis to explore whether early detection could explain this difference in academic outcomes.

### Mediation analysis

A mediation analysis was conducted to test whether early detection, measured by grade level at first outpatient visit, mediated the relationship between comorbid ADHD status and academic performance. For reading delay, ADHD status significantly predicted earlier detection (a = −1.04, p = 0.001), but early detection did not significantly predict reading outcomes (b = −0.06, p = 0.125). The direct effect of ADHD status on reading delay remained statistically significant (c′ = 0.47, p = 0.018), suggesting that early detection alone could not explain the better reading performance. This implies that other factors, possibly neurocognitive differences, may contribute to such an advantage. (Fig 1)

**Fig 1:**
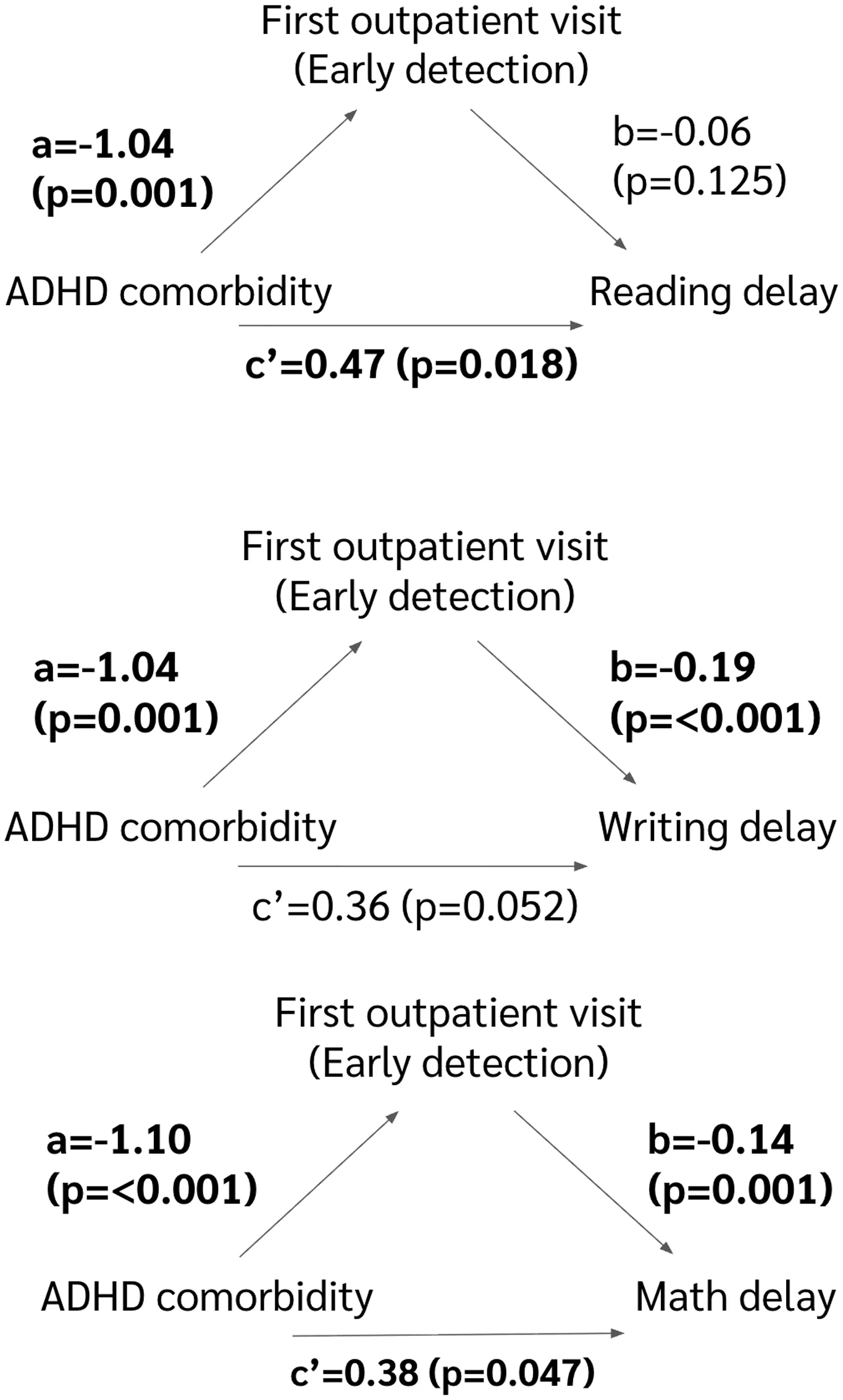
Mediation analysis between ADHD comorbidity, early detection, reading (upper) / writing (middle) / math (lower) delays.

For writing delay, ADHD status significantly predicted earlier detection (a = −1.04, p = 0.001). In this case, early detection was significantly associated with less severe writing delay (b = −0.19, p < 0.001). The direct effect of ADHD status on writing delay was not significant after accounting for early detection (c′ = 0.36, p = 0.052), indicating that early detection fully mediated the relationship between ADHD and writing outcomes (Fig 1).

For math delay, both the indirect and direct pathways were statistically significant. ADHD status predicted earlier detection (a = −1.10, p < 0.001) and earlier detection predicted better math outcomes (b = −0.14, p = 0.001). The direct effect of ADHD on math delay remained significant (c′ = 0.38, p = 0.047), suggesting that both early detection and other contributing factors may play a role in explaining the lower math delays observed in children with SLD and comorbid ADHD (Fig 1).

## Discussion

In this study, we compared children with specific learning disorder (SLD) alone to those with comorbid attention-deficit/hyperactivity disorder (ADHD) and found differences in both timing of detection and severity of academic delays. Children with both SLD and ADHD were diagnosed at a younger grade level and exhibited significantly less severe delays in reading, writing, and mathematics than those with SLD alone. These findings showed that SLD with ADHD comorbidity and SLD alone have different entities and characteristics. Our study also challenges the common assumption that ADHD with comorbidity usually leads to worse functional impairment [7].

Our findings which revealed that children with SLD and ADHD comorbidity were detected and referred almost one year earlier compared to the SLD alone group are supported by prior research. The studies highlighted the earlier onset and visibility of ADHD symptoms, particularly due to behavioral and inattention concerns. ADHD often leads to early school referrals and identification because symptoms are disruptive and easily observed by parents and teachers [8], potentially introducing referral and ascertainment biases. In contrast, learning disorders such as dyslexia and dysgraphia are often identified later once children begin to fail reading or writing tasks, which tend to be more complex in upper elementary grades [9].

One may speculate that early detection of SLD with ADHD comorbidity accounts for the less severe delay in reading, writing, and mathematics. Thus, we utilized mediation analysis by putting the ADHD factor and the early detection (first OPD visit) factor in the same model. Our mediation analysis revealed that children with SLD and comorbid ADHD were more likely to be diagnosed at an earlier grade level, and poor writing skills are influenced mainly by early detection of ADHD. Writing involves fine motor control, orthographic working memory, and graphomotor skills, which include posterior parietal and cerebellar networks involved in handwriting coordination and fluency [10]. In ADHD patients, writing problems may be attributed to poor coordination and attention in the learning process [11]. As such, early detection of ADHD may play a huge role in improving writing outcomes by allowing children to receive timely support.

On the contrary, early detection did not fully mediate the relationship between ADHD comorbidity and reading or math delays, suggesting that other neurobiological mechanisms might contribute to these differences. One possible hypothesis is that the neural circuit in ADHD might counteract the circuit in reading and math disabilities. For instance, dyslexia is associated with atypical activation in the left occipitotemporal and temporoparietal regions, including the visual word form and phonological processing areas that are crucial for reading fluently [12]. Dyscalculia involves dysfunction in parietal and frontal circuits responsible for number sense and working memory [13]. For ADHD, it is known to affect the frontoparietal attentional networks which are brain areas that overlap in reading for dyslexia and mathematics for dyscalculia [14]. Shared neural circuits may interact in ways that alter how academic skills develop in children with both conditions.

According to Parks et al. [15], reading ability in ADHD is highly variable from poor to above average. This supports the idea that not all children with ADHD exhibit reading impairment. Some demonstrate decoding and comprehension skills at a similar level as their neurotypical peers. Similarly, Wolf [16] also shows how while the inattentive subtype has difficulty sustaining attention, some may present with enhanced abilities in systemizing and focused information processing. The ability to focus on specific interests, strong retention of numerical information, and an aptitude for recognizing complex patterns may enable an above average performance in number tasks.

Furthermore, we hypothesize that the tendency of some children with ADHD to approach tasks impulsively, such as reading without overthinking, may bypass the hesitation and anxiety commonly seen in children with SLD alone. In math, children with ADHD may show confidence in quick estimation which, while sometimes prone to errors, can help them perform better on timed tests. Future studies could benefit from analyzing which cognitive abilities of children in the comorbid group are associated with protective effects in specific academic domains.

Together, these findings suggest that while early detection is an important factor, it cannot fully explain the advantage in children with comorbid ADHD. Neurocognitive differences across learning domains may underlie the distinct patterns seen in reading, writing, and math abilities. This result emphasizes the role of early detection and possibly unique neurobiological mechanisms in these children. However, these proposed mechanisms remain speculative and require further investigation. Despite the high rate of comorbidity between SLD and ADHD, no prior study has directly compared the severity of reading, writing, and math delays between SLD only and comorbid groups. This gap makes our study unique by offering a comprehensive comparison across all three academic domains, providing valuable insight into different developmental profiles of children with learning disabilities.

## Strengths & limitations

To our knowledge, our study is among the first studies to directly compare the severity of reading, writing, and math delays between children with SLD alone and those with comorbid ADHD. Previous research has focused largely on comorbidity rates or overlapping symptoms, but our study compares literacy outcomes across major domains. In addition, we controlled important variables by including both IQ scores and developmental risk factors (preterm birth, neonatal complications, history of seizures, or speech delay) in our analysis. This allowed for a more nuanced interpretation of the differences between groups.

However, there are some limitations to consider. Our retrospective cross-sectional design limits causality, as temporal relationships between ADHD comorbidity, early detection, and academic outcomes cannot be established. Additionally, existing medical records may contain incomplete data collection and introduce information bias. A longitudinal cohort design with follow-up assessments would be valuable to determine how ADHD comorbidity influences future academic outcomes, and whether early detection mediates literacy skill development over time.

## Conclusion & clinical implications

In summary, this study found that children with SLD and comorbid ADHD were diagnosed at an earlier grade level and showed less severe delays in reading, writing, and math compared to those with SLD alone. Mediation analysis revealed that early detection explained the advantage in writing skills but not fully for reading and math, suggesting that other neurocognitive factors may be involved. These findings challenge the common assumption that ADHD comorbidity automatically worsens learning outcomes and instead highlight how early recognition and referral, often prompted by behaviors related to ADHD, may promote better academic performance in some domains.

Clinically, the results support the importance of early identification as it allows for timely academic intervention and may also result in less severe learning delays, especially in writing. Additionally, this raises the possibility that children with SLD without ADHD may experience greater delays because they lack the more visible behavioral symptoms that prompt referral. Therefore, implementing systematic screening programs during early elementary years and increasing awareness of early warning signs among parents and teachers may help promote earlier diagnosis and timely intervention in this population.

## References

[1] Pham AV, Riviere A. Specific learning disorders and ADHD: Current issues in diagnosis across clinical and educational settings. Curr Psychiatry Rep. 2015;17(6):38. doi: 10.1007/s11920-015-0584-y 25894357

[2] Yusriyyah QN, Aziz ARH, Setiawati Y, Dianasari D, Pradanita VN, Ardani IGAI. Learning disorder in attention deficit hyperactivity disorder (ADHD) children: A literature review. Int J Sci Adv. 2023;4(1). doi: 10.51542/ijscia.v4i1.4

[3] DuPaul GJ, Gormley MJ, Laracy SD. Comorbidity of LD and ADHD. J Learn Disabil. 2012;46(1):43–51. doi: 10.1177/0022219412464351 23144063

[4] DuPaul GJ, Volpe RJ. ADHD and learning disabilities: Research findings and clinical implications. Curr Atten Disord Rep. 2009;1(4):152–5. doi: 10.1007/s12618-009-0021-4

[5] Suphasawat A, Hongsanguansri S, Seree P, Rotjananirunkit O. The study of internet use and academic achievement of elementary students in Bangkok. J Educ Dev Psychol. 2016;6(2):71–83. doi: 10.5539/jedp.v6n2p71

[6] Yasumitsu-Lovell K, Thompson L, Fernell E, Eitoku M, Suganuma N, Gillberg C, et al. Japan environment and children’s study group. Pre-/perinatal reduced optimality and neurodevelopment at 1 month and 3 years of age: Results from the Japan Environment and Children’s Study (JECS). PLoS One. 2023;18(1):e0280249. doi: 10.1371/journal.pone.0280249 PMC9833583 36630408

[7] Smith T, Adams G. The effect of comorbid AD/HD and learning disabilities on parent-reported behavioral and academic outcomes of children. Learn Disabil Q. 2006;29(2):101–12. doi: 10.2307/30035538

[8] Rocco I, Corso B, Bonati M, Minicuci N. Time of onset and/or diagnosis of ADHD in European children: A systematic review. BMC Psychiatry. 2021;21(1):575. doi: 10.1186/s12888-021-03547-x 34784913 PMC8594188

[9] Thompson PA, Hulme C, Nash HM, Gooch D, Hayiou-Thomas E, Snowling MJ. Developmental dyslexia: predicting individual risk. J Child Psychol Psychiatry. 2015;56(9):976–87. doi: 10.1111/jcpp.12412 25832320 PMC4672694

[10] Vlachos F, Avramidis E. The difference between developmental dyslexia and dysgraphia: Recent neurobiological evidence. Int J Neurosci Behav Sci. 2020;8(1):1–5. doi: 10.13189/ijnbs.2020.080101

[11] Mokobane M, Pillay BJ, Meyer A. Fine motor deficits and attention deficit hyperactivity disorder in primary school children. S Afr J Psychiatr. 2019;25:1232. doi: 10.4102/sajpsychiatry.v25i0.1232 30899581 PMC6424539

[12] Habib M. The neurological basis of developmental dyslexia: An overview and working hypothesis. Brain. 2000;123(12):2373–99. doi: 10.1093/brain/123.12.2373 11099442

[13] Butterworth B, Varma S, Laurillard D. Dyscalculia: From brain to education. Science. 2011;332(6033):1049–53. doi: 10.1126/science.1201536 21617068

[14] Yerys BE, Tunç B, Satterthwaite TD, Antezana L, Mosner MG, Bertollo JR, et al. Functional connectivity of frontoparietal and salience/ventral attention networks have independent associations with co-occurring attention-deficit/hyperactivity disorder symptoms in children with autism. Biol Psychiatry Cogn Neurosci Neuroimaging. 2019;4(4):343–51. doi: 10.1016/j.bpsc.2018.12.012 30777604 PMC6456394

[15] Parks KMA, Moreau CN, Hannah KE, Brainin L, Joanisse MF. The task matters: A scoping review on reading comprehension abilities in ADHD. J Atten Disord. 2022;26(10):1304–24. doi: 10.1177/10870547211068047 34961391

[16] Wolf CJ. Why are some people with ADHD good at math and others bad? Exploring cognitive profiles, working memory differences, autistic-leaning traits, and emotional/dopaminergic factors. 2025 [cited 2026 Feb 19]. Available from: https://www.researchgate.net/publication/392313055_Why_Are_Some_People_with_ADHD_Good_at_Math_and_Others_Bad_Exploring_cognitive_profiles_working_memory_differences_autistic-leaning_traits_and_emotionaldopaminergic_factors

