# Supplementary material for: The ADHD advantage: Comorbid attention-deficit/hyperactivity disorder is associated with earlier diagnosis and better academic outcomes in children with specific language disorder
Source: PLoS One. 2026 Oct 5;21(10):e0356489. doi: 10.1371/journal.pone.0356489 (PMC13638033; doi:10.1371/journal.pone.0356489)
Supplement: Supplementary file 1 [file pone.0356489.s002.docx]

**Our Responses to the Reviewers**

Thank you for reviewing our manuscript. We have revised our explanations as suggested and changed to the yellow highlighted texts as attached.

Reviewer #1: I have some comments which I hope will help improve the manuscript.

1) In lines 37-38, it is said that "individuals typically have an average IQ, loosely estimated around 75 (plus/minus) 5", but 75 is not an average IQ.

→ Our revision (line 37-38): Affected individuals are expected to have intellectual functioning within normal range [1].

2) The Methods section says that inclusion criteria included complete data on, among others, "achievement tests, and literacy skill assessments". Which tests or psychometrical tools were used to perform that assessment? They should be described in the Measures subsection.

3) In the same vein, the Measures subsection says that "the severity of learning disorder was evaluated using standardized achievement tests". Again, I would ask which tests were used. The reference provided does not seem a direct reference to any of such tests.

→ Our revision for 2) & 3) : Add descriptions of the achievement test in line 123-133. Remove “literacy skill assessments” as the data were not analyzed or reported in this study.

4) Line 160 mentions a total risk score calculated from birth risks, history of seizures, and histor of speech delay. One wonders why that score was calculated, since using the variables separately seemed to have given good results and the risk score did not add any value to this research.

→ In our study, the frequency of each risk factor was relatively low. We have performed a separate analysis of each risk factor and found no significant associations. We then decided to use a cumulative risk score to increase the sensitivity for detecting any associations with neurodevelopmental disorders as suggested by our referenced study [6] (line 167-168), although there was still no significant difference in cumulative risk score between our two groups.

Reviewer #2: The manuscript addresses an important and clinically relevant topic regarding the impact of comorbid ADHD on the identification and severity of specific learning disorders (SLD). The study includes a reasonably sized clinical sample and provides useful insights into the relationship between ADHD, timing of diagnosis, and academic outcomes in children with SLD.

The manuscript is generally well organized, clearly written, and the methodology is appropriate for the research questions. The statistical analyses are suitable and the results are presented clearly. The findings that children with comorbid ADHD are identified earlier and demonstrate less severe academic delays than children with SLD alone are interesting and have potential implications for clinical screening and educational interventions.

I have a few minor suggestions that may strengthen the manuscript:

5) The discussion would benefit from additional consideration of potential referral and ascertainment biases. Children with ADHD may come to clinical attention earlier because of behavioral symptoms, which could contribute to earlier identification of learning difficulties.

→ Revision: added your suggestion in line 235-236

6) Although the authors discuss possible neurobiological mechanisms, these explanations were not directly examined in the current study. It may be helpful to emphasize that these interpretations remain speculative and require further investigation.

→ Revision: added your suggestion in line 291-292

7) Additional discussion regarding the clinical implications of earlier identification, including potential strategies for improving early detection of SLD in children without ADHD, would enhance the practical relevance of the findings.

→ Revision: added your suggestion in the clinical implication section (Line 330-336)

8) The limitations section could further emphasize the cross-sectional and retrospective nature of the study, which limits causal inference.

→ Revision: added your suggestion in line 309-313

Overall, this is a well-conducted study that contributes meaningful information to the literature on learning disorders and ADHD. The manuscript is suitable for publication after minor revisions.
