# Supplementary material for: The ADHD advantage: Comorbid attention-deficit/hyperactivity disorder is associated with earlier diagnosis and better academic outcomes in children with specific language disorder
Source: PLoS One. 2026 Oct 5;21(10):e0356489. doi: 10.1371/journal.pone.0356489 (PMC13638033; doi:10.1371/journal.pone.0356489)
Supplement: Peer review history

# Peer review: Decision Letter 0

**DOI:** 10.1371/journal.pone.0356489.r001

**Authors:** Andrea Martinuzzi

9 Jun 2026

Dear Dr. Thongpan,

Thank you for submitting your manuscript to PLOS ONE. After careful consideration, we feel that it has merit but does not fully meet PLOS ONE’s publication criteria as it currently stands. Therefore, we invite you to submit a revised version of the manuscript that addresses the points raised during the review process.

plosone@plos.org. When you're ready to submit your revision, log on to https://www.editorialmanager.com/pone/ and select the 'Submissions Needing Revision' folder to locate your manuscript file.

- A letter that responds to each point raised by the academic editor and reviewer(s). You should upload this letter as a separate file labeled 'Response to Reviewers'.
- A marked-up copy of your manuscript that highlights changes made to the original version. You should upload this as a separate file labeled 'Revised Manuscript with Track Changes'.
- An unmarked version of your revised paper without tracked changes. You should upload this as a separate file labeled 'Manuscript'.

If you would like to make changes to your financial disclosure, please include your updated statement in your cover letter. Guidelines for resubmitting your figure files are available below the reviewer comments at the end of this letter.

If applicable, we recommend that you deposit your laboratory protocols in protocols.io to enhance the reproducibility of your results. Protocols.io assigns your protocol its own identifier (DOI) so that it can be cited independently in the future. For instructions see: https://journals.plos.org/plosone/s/submission-guidelines#loc-laboratory-protocols. Additionally, PLOS ONE offers an option for publishing peer-reviewed Lab Protocol articles, which describe protocols hosted on protocols.io. Read more information on sharing protocols at https://plos.org/protocols?utm_medium=editorial-email&utm_source=authorletters&utm_campaign=protocols.

As the corresponding author, your ORCID iD is verified in the submission system and will appear in the published article. PLOS supports the use of ORCID, and we encourage all coauthors to register for an ORCID iD and use it as well. Please encourage your coauthors to verify their ORCID iD within the submission system before final acceptance, as unverified ORCID iDs will not appear in the published article. *Only*  the individual author can complete the verification step; PLOS staff *cannot*  verify ORCID iDs on behalf of authors.

We look forward to receiving your revised manuscript.

Kind regards,

Andrea Martinuzzi

Academic Editor

PLOS One

Journal Requirements:

When submitting your revision, we need you to address these additional requirements.

1. Please ensure that your manuscript meets PLOS ONE's style requirements, including those for file naming. The PLOS ONE style templates can be found at

https://journals.plos.org/plosone/s/file?id=wjVg/PLOSOne_formatting_sample_main_body.pdf and

https://journals.plos.org/plosone/s/file?id=ba62/PLOSOne_formatting_sample_title_authors_affiliations.pdf.

2. In the online submission form you indicate that your data is not available for proprietary reasons and have provided a contact point for accessing this data. Please note that your current contact point is a co-author on this manuscript. According to our Data Policy, the contact point must not be an author on the manuscript and must be an institutional contact, ideally not an individual. Please revise your data statement to a non-author institutional point of contact, such as a data access or ethics committee, and send this to us via return email. Please also include contact information for the third party organization, and please include the full citation of where the data can be found.

3. If the reviewer comments include a recommendation to cite specific previously published works, please review and evaluate these publications to determine whether they are relevant and should be cited. There is no requirement to cite these works unless the editor has indicated otherwise.

4. Please review your reference list to ensure that it is complete and correct. If you have cited papers that have been retracted, please include the rationale for doing so in the manuscript text, or remove these references and replace them with relevant current references. Any changes to the reference list should be mentioned in the rebuttal letter that accompanies your revised manuscript. If you need to cite a retracted article, indicate the article’s retracted status in the References list and also include a citation and full reference for the retraction notice.

[Note: HTML markup is below. Please do not edit.]

Reviewers' comments:

Reviewer's Responses to Questions

**Comments to the Author**

1. Is the manuscript technically sound, and do the data support the conclusions?

Reviewer #1: Yes

Reviewer #2: Yes

**********

2. Has the statistical analysis been performed appropriately and rigorously?

Reviewer #1: Yes

Reviewer #2: Yes

**********

3. Have the authors made all data underlying the findings in their manuscript fully available?

The PLOS Data policy

Reviewer #1: No

Reviewer #2: No

**********

4. Is the manuscript presented in an intelligible fashion and written in standard English?

Reviewer #1: Yes

Reviewer #2: Yes

**********

Reviewer #1: I have sme comments which I hope will help improve the manuscript.

1. In lines 37-38, it is said that "individuals typically have an average IQ, loosely estimated around 75 (plus/minus) 5", but 75 is not an average IQ.

2. The Methods section says that inclusion criteria included complete data on, among others, "achievement tests, and literacy skill assessments". Which tests or psychometrical tools were used to perform that assessment? They should be described in the Measures subsection.

3. In the same vein, the Measures subsection says tht "the severity of learning disorder was evaluated using standardized achievment tests". Again, I would ask which tests were used. The reference provided does not seem a direct reference to any of such tests.

4. Line 160 mentions a total risk score calculated from birth risks, history of seizures, and histor of speech delay. One wonders why that score was calculated, since using the variables separately seemed to have given good results and the risk score did not add any value to this research.

Reviewer #2: The manuscript addresses an important and clinically relevant topic regarding the impact of comorbid ADHD on the identification and severity of specific learning disorders (SLD). The study includes a reasonably sized clinical sample and provides useful insights into the relationship between ADHD, timing of diagnosis, and academic outcomes in children with SLD.

The manuscript is generally well organized, clearly written, and the methodology is appropriate for the research questions. The statistical analyses are suitable and the results are presented clearly. The findings that children with comorbid ADHD are identified earlier and demonstrate less severe academic delays than children with SLD alone are interesting and have potential implications for clinical screening and educational interventions.

I have a few minor suggestions that may strengthen the manuscript:

The discussion would benefit from additional consideration of potential referral and ascertainment biases. Children with ADHD may come to clinical attention earlier because of behavioral symptoms, which could contribute to earlier identification of learning difficulties.

Although the authors discuss possible neurobiological mechanisms, these explanations were not directly examined in the current study. It may be helpful to emphasize that these interpretations remain speculative and require further investigation.

Additional discussion regarding the clinical implications of earlier identification, including potential strategies for improving early detection of SLD in children without ADHD, would enhance the practical relevance of the findings.

The limitations section could further emphasize the cross-sectional and retrospective nature of the study, which limits causal inference.

Overall, this is a well-conducted study that contributes meaningful information to the literature on learning disorders and ADHD. The manuscript is suitable for publication after minor revisions.

**********

what does this mean?). If published, this will include your full peer review and any attached files.

If you choose “no”, your identity will remain anonymous but your review may still be made public.

**Do you want your identity to be public for this peer review?**  For information about this choice, including consent withdrawal, please see our Privacy Policy

Reviewer #1: No

Reviewer #2: **Yes:**  VENKATA VIJAYA K DALAI

**********

[NOTE: If reviewer comments were submitted as an attachment file, they will be attached to this email and accessible via the submission site. Please log into your account, locate the manuscript record, and check for the action link "View Attachments". If this link does not appear, there are no attachment files.]

To ensure your figures meet our technical requirements, please review our figure guidelines: https://journals.plos.org/plosone/s/figures

You may also use PLOS’s free figure tool, NAAS, to help you prepare publication quality figures: https://journals.plos.org/plosone/s/figures#loc-tools-for-figure-preparation.

NAAS will assess whether your figures meet our technical requirements by comparing each figure against our figure specifications.

# Author comment: Author response to Decision Letter 1

**DOI:** 10.1371/journal.pone.0356489.r002

20 Jul 2026

Our Responses to the Reviewers

Thank you for reviewing our manuscript. We have revised our explanations as suggested and changed to the yellow highlighted texts as attached.

Reviewer #1: I have some comments which I hope will help improve the manuscript.

1) In lines 37-38, it is said that "individuals typically have an average IQ, loosely estimated around 75 (plus/minus) 5", but 75 is not an average IQ.

→ Our revision (line 37-38): Affected individuals are expected to have intellectual functioning within normal range [1].

2) The Methods section says that inclusion criteria included complete data on, among others, "achievement tests, and literacy skill assessments". Which tests or psychometrical tools were used to perform that assessment? They should be described in the Measures subsection.

3) In the same vein, the Measures subsection says that "the severity of learning disorder was evaluated using standardized achievement tests". Again, I would ask which tests were used. The reference provided does not seem a direct reference to any of such tests.

→ Our revision for 2) & 3) : Add descriptions of the achievement test in line 123-133. Remove “literacy skill assessments” as the data were not analyzed or reported in this study.

4) Line 160 mentions a total risk score calculated from birth risks, history of seizures, and histor of speech delay. One wonders why that score was calculated, since using the variables separately seemed to have given good results and the risk score did not add any value to this research.

→ In our study, the frequency of each risk factor was relatively low. We have performed a separate analysis of each risk factor and found no significant associations. We then decided to use a cumulative risk score to increase the sensitivity for detecting any associations with neurodevelopmental disorders as suggested by our referenced study [6] (line 167-168), although there was still no significant difference in cumulative risk score between our two groups.

Reviewer #2: The manuscript addresses an important and clinically relevant topic regarding the impact of comorbid ADHD on the identification and severity of specific learning disorders (SLD). The study includes a reasonably sized clinical sample and provides useful insights into the relationship between ADHD, timing of diagnosis, and academic outcomes in children with SLD.

The manuscript is generally well organized, clearly written, and the methodology is appropriate for the research questions. The statistical analyses are suitable and the results are presented clearly. The findings that children with comorbid ADHD are identified earlier and demonstrate less severe academic delays than children with SLD alone are interesting and have potential implications for clinical screening and educational interventions.

I have a few minor suggestions that may strengthen the manuscript:

5) The discussion would benefit from additional consideration of potential referral and ascertainment biases. Children with ADHD may come to clinical attention earlier because of behavioral symptoms, which could contribute to earlier identification of learning difficulties.

→ Revision: added your suggestion in line 235-236

6) Although the authors discuss possible neurobiological mechanisms, these explanations were not directly examined in the current study. It may be helpful to emphasize that these interpretations remain speculative and require further investigation.

→ Revision: added your suggestion in line 291-292

7) Additional discussion regarding the clinical implications of earlier identification, including potential strategies for improving early detection of SLD in children without ADHD, would enhance the practical relevance of the findings.

→ Revision: added your suggestion in the clinical implication section (Line 330-336)

8) The limitations section could further emphasize the cross-sectional and retrospective nature of the study, which limits causal inference.

→ Revision: added your suggestion in line 309-313

Overall, this is a well-conducted study that contributes meaningful information to the literature on learning disorders and ADHD. The manuscript is suitable for publication after minor revisions.

Attachment Submitted filename: response to reviewers.docx

# Peer review: Decision Letter 1

**DOI:** 10.1371/journal.pone.0356489.r003

**Authors:** Andrea Martinuzzi

4 Aug 2026

The ADHD advantage: comorbid attention-deficit/hyperactivity disorder is associated with earlier diagnosis and better academic outcomes in children with specific language disorder

PONE-D-26-10865R1

Dear Dr. Thongpan,

We’re pleased to inform you that your manuscript has been judged scientifically suitable for publication and will be formally accepted for publication once it meets all outstanding technical requirements.

Within one week, you’ll receive an e-mail detailing the required amendments. When these have been addressed, you’ll receive a formal acceptance letter and your manuscript will be scheduled for publication.

An invoice will be generated when your article is formally accepted. Please note, if your institution has a publishing partnership with PLOS and your article meets the relevant criteria, all or part of your publication costs will be covered. Please make sure your user information is up-to-date by logging into Editorial Manager at Editorial Manager® and clicking the ‘Update My Information' link at the top of the page. For questions related to billing, please contact billing support.

If your institution or institutions have a press office, please notify them about your upcoming paper to help maximize its impact. If they’ll be preparing press materials, please inform our press team as soon as possible -- no later than 48 hours after receiving the formal acceptance. Your manuscript will remain under strict press embargo until 2 pm Eastern Time on the date of publication. For more information, please contact onepress@plos.org.

Kind regards,

Andrea Martinuzzi

Academic Editor

PLOS One

Additional Editor Comments (optional):

Reviewers' comments:

Reviewer's Responses to Questions

**Comments to the Author**

Reviewer #1: All comments have been addressed

Reviewer #2: All comments have been addressed

**********

2. Is the manuscript technically sound, and do the data support the conclusions?

Reviewer #1: Yes

Reviewer #2: Yes

**********

3. Has the statistical analysis been performed appropriately and rigorously?

Reviewer #1: Yes

Reviewer #2: Yes

**********

4. Have the authors made all data underlying the findings in their manuscript fully available?

The PLOS Data policy

Reviewer #1: Yes

Reviewer #2: No

**********

5. Is the manuscript presented in an intelligible fashion and written in standard English?

Reviewer #1: Yes

Reviewer #2: Yes

**********

Reviewer #1: (No Response)

Reviewer #2: Thank you for the thorough revision of the manuscript. The authors have adequately addressed all of the comments raised during the previous round of review. The revisions have improved the clarity of the methodology, strengthened the discussion by acknowledging referral bias and study limitations, and appropriately tempered the interpretation of the findings. The manuscript is technically sound, well written, and the conclusions are supported by the presented data. I have no further comments and believe the manuscript is suitable for publication in its current form.

**********

what does this mean?). If published, this will include your full peer review and any attached files.

If you choose “no”, your identity will remain anonymous but your review may still be made public.

**Do you want your identity to be public for this peer review?**  For information about this choice, including consent withdrawal, please see our Privacy Policy

Reviewer #1: No

Reviewer #2: **Yes:**  VENKATA VIJAYA KUMAR DALAI

**********

# Editor report: Acceptance letter

**DOI:** 10.1371/journal.pone.0356489.r004

**Authors:** Andrea Martinuzzi

PONE-D-26-10865R1

PLOS One

Dear Dr. Thongpan,

I'm pleased to inform you that your manuscript has been deemed suitable for publication in PLOS One. Congratulations! Your manuscript is now being handed over to our production team.

At this stage, our production department will prepare your paper for publication. This includes ensuring the following:

* All references, tables, and figures are properly cited

* All relevant supporting information is included in the manuscript submission,

* There are no issues that prevent the paper from being properly typeset

You will receive further instructions from the production team, including instructions on how to review your proof when it is ready. Please keep in mind that we are working through a large volume of accepted articles, so please give us a few days to review your paper and let you know the next and final steps.

Lastly, if your institution or institutions have a press office, please let them know about your upcoming paper now to help maximize its impact. If they'll be preparing press materials, please inform our press team within the next 48 hours. Your manuscript will remain under strict press embargo until 2 pm Eastern Time on the date of publication. For more information, please contact onepress@plos.org.

You will receive an invoice from PLOS for your publication fee after your manuscript has reached the completed accept phase. If you receive an email requesting payment before acceptance or for any other service, this may be a phishing scheme. Learn how to identify phishing emails and protect your accounts at https://explore.plos.org/phishing.

If we can help with anything else, please email us at customercare@plos.org.

Thank you for submitting your work to PLOS One and supporting open access.

Kind regards,

PLOS One Editorial Office Staff

on behalf of

Dr. Andrea Martinuzzi

Academic Editor

PLOS One
